# Clinical Application of Heparin-Conjugated Fibrin Hydrogel in the Treatment of Osteochondral Defects of the Talus: Preliminary Results

**DOI:** 10.3390/biomedicines14061398

**Published:** 2026-06-21

**Authors:** Dina Saginova, Meruyert Makhmetova, Yerik Raimagambetov, Bagdat Balbossynov, Vyacheslav Ogay, Ulunay Kanatli

**Affiliations:** 1 National Scientific Center of Traumatology and Orthopedics named after Academician N. D. Batpenov, Astana 010000, Kazakhstan; 2 Research School, Astana Medical University, Astana 010000, Kazakhstan; 3 Stem Cell Laboratory, National Center for Biotechnology, Astana 010000, Kazakhstan; 4 Faculty of Medicine, Gazi University, Ankara 06560, Turkey

**Keywords:** osteochondral lesion of the talus, cartilage regeneration, heparin-conjugated fibrin hydrogel, mesenchymal stromal cells, arthroscopy, tissue engineering, regenerative medicine

## Abstract

**Background**: Osteochondral lesions of the talus (OLT) remain a challenging condition due to the limited regenerative potential of articular cartilage. Conventional bone marrow stimulation (BMS) techniques often result in fibrocartilage formation with inferior biomechanical properties. This study aimed to evaluate the safety and preliminary clinical efficacy of an arthroscopically assisted, single-stage injection of a heparin-conjugated fibrin hydrogel (HCFH) for OLT treatment. **Methods**: Twelve patients with symptomatic OLT underwent arthroscopic debridement, microfracturing, and HCFH injection containing autologous mesenchymal stromal cells (MSCs) and growth factors. Safety was assessed through systematic monitoring of adverse events (graded according to Common Terminology Criteria for Adverse Events criteria), wound healing, and serial laboratory inflammatory markers (leukocytes, erythrocyte sedimentation rate, C-reactive protein) during early and late follow-up. Clinical outcomes were evaluated using the Visual Analog Scale (VAS) and American Orthopedic Foot and Ankle Society score (AOFAS) preoperatively and at 6 and 12 months. Morphological assessment was performed using magnetic resonance imaging (MRI) with the modified Magnetic Resonance Observation of Cartilage Repair Tissue (MOCART) scoring system, evaluated independently by two blinded musculoskeletal radiologists. **Results**: No serious adverse events (Grade III–IV) were observed during the 12-month follow-up. All adverse events were mild (Grade I) and self-limited. A transient postoperative elevation in inflammatory markers was observed, returning to clinically acceptable levels by day 14. Significant improvements were noted in pain (VAS decreased from 6.0 to 2.0) and ankle function (AOFAS increased from 70.0 to 90.6) (*p* < 0.001). MRI demonstrated progressive morphological improvement, with the MOCART score increasing from 34.16 ± 17.1 at 6 months to 75 ± 5.43 at 12 months (*p* < 0.001). This increase corresponded with imaging features consistent with tissue maturation over time. The favorable MOCART outcomes observed in this study may be explained by the regenerative properties of heparin-conjugated fibrin hydrogels; however, larger randomized controlled trials with longer follow-up are needed to confirm the durability of the regenerated tissue. Interobserver agreement was substantial to almost perfect for MOCART scoring (κ = 0.68–0.84), with perfect agreement observed for surface assessment, bony defect/overgrowth, and cysts. **Conclusions**: Within the limitations of this study, single-stage HCFH injection demonstrated an acceptable safety profile and favorable preliminary clinical and radiological outcomes at 12 months. These findings suggest potential regenerative capability; however, controlled studies with larger cohorts and longer follow-up are required to determine comparative efficacy and long-term durability.

## 1. Introduction

Osteochondral lesions of the talus (OLT) are defects involving both the articular cartilage and the subchondral bone [1,2]. These injuries commonly arise following trauma—such as fractures, dislocations, or chronic ankle instability—and initiate a cascade of degenerative changes that progressively damage the joint surface. Ankle sprains represent the most common sports injury, comprising approximately 15–20% of all sports-related traumas, with osteochondral damage of the talus observed in up to 50% of such cases [3,4]. Without appropriate intervention, OLTs may enlarge and lead to irreversible cartilage loss and early osteoarthritis.

Conventional surgical methods remain largely symptomatic and fail to restore the structural and functional properties of native hyaline cartilage. Techniques aimed at stimulating endogenous repair—such as microfracture, drilling, or abrasion arthroplasty—induce the release of mesenchymal stromal cells (MSCs) and growth factors from the subchondral bone [5,6,7,8]. However, these procedures typically result in the potential formation of fibrocartilaginous tissue, which is biomechanically inferior and prone to degeneration.

Osteochondral autograft transplantation (OATS) is another well-established approach that replaces the damaged cartilage area with mature hyaline tissue supported by a subchondral bone base [9,10,11,12]. Cylindrical grafts are harvested from a low-load region of the knee joint and injected into the talar defect in a mosaic-like pattern, allowing restoration of the articular contour and load-bearing capacity. Despite its advantages, OATS is limited by the small amount of available donor material, the risk of donor-site morbidity, and the technical challenges of achieving full surface congruency.

Cell-based techniques for the treatment of cartilage defects, including Autologous Chondrocyte Implantation (ACI), Matrix-Induced Autologous Chondrocyte Implantation (MACI), Autologous Matrix-Induced Chondrogenesis (AMIC), and Bone Marrow-Derived Cell Transplantation (BMDCT), are designed to stimulate cartilage regeneration. Among these approaches, ACI and MACI have demonstrated the most favorable clinical outcomes in the treatment of medium- and large-sized defects, with success rates of up to 80–90% and the formation of hyaline-like cartilage [13]. The AMIC technique involves the use of biocompatible three-dimensional matrices that serve as a scaffold to stabilize the bone marrow clot and support chondrogenic differentiation of bone marrow-derived cells.

However, in Kazakhstan the clinical implementation of these technologies remains limited. In routine orthopedic practice, bone marrow stimulation (BMS) techniques and autologous osteochondral transplantation are currently the most commonly used treatment methods. The use of ACI and MACI is constrained by the high cost of treatment, the need for two-stage surgical procedures, and the requirement for specialized GMP-compliant cell culture laboratories for chondrocyte isolation and expansion. Similar regulatory and manufacturing requirements also apply to expanded MSC-based therapies. However, MSC-based approaches may offer practical advantages related to higher proliferative capacity, less invasive tissue harvesting procedures, and broader scalability for clinical manufacturing. In addition, the biocompatible matrices required for the AMIC technique are not available in Kazakhstan, which further restricts the use of this approach.

Given these constraints, the development of safe, cost-effective, and durable regenerative methods remains a priority. Recent advances in regenerative orthopedics have highlighted the potential of injectable biocomposite hydrogels—particularly those based on heparin-conjugated fibrin matrices incorporating MSCs and growth factors—to create a localized three-dimensional microenvironment mimicking the extracellular cartilage matrix [14,15,16].

Hydrogels have emerged as one of the most promising biomaterial platforms for cartilage tissue engineering due to their high water content, biocompatibility, and ability to mimic the native extracellular matrix microenvironment [17]. Injectable hydrogels can provide structural support for encapsulated cells, facilitate nutrient diffusion, and enable localized delivery of bioactive factors that promote chondrogenesis and tissue repair [18]. Natural hydrogels based on fibrin, collagen, hyaluronic acid, and gelatin have demonstrated favorable biological properties and have been widely investigated for cartilage regeneration [19]. However, challenges remain regarding their mechanical stability, integration with host tissue, and long-term durability in load-bearing joints. Recent advances in hydrogel engineering, including growth factor-binding modifications such as heparin conjugation, have been developed to improve scaffold performance and enhance regenerative outcomes [20].

Heparin conjugation plays a key role in regulating the biological performance of fibrin-based hydrogels. Heparin is a highly sulfated glycosaminoglycan that exhibits strong electrostatic affinity for numerous heparin-binding growth factors, including members of the transforming growth factor-β (TGF-β) families [21]. When covalently conjugated to fibrinogen, heparin introduces specific binding domains within the fibrin network that enable sequestration and stabilization of these bioactive molecules [22,23]. This interaction protects growth factors from rapid diffusion and enzymatic degradation while enabling controlled and sustained release from the hydrogel matrix [20]. As a result, the local concentration and bioavailability of growth factors at the defect site can be maintained for a prolonged period compared with conventional fibrin hydrogels, which typically exhibit rapid growth factor diffusion and short biological activity [24].

In addition, fibrin network provides a favorable three-dimensional microenvironment for mesenchymal stromal cells, supporting cell adhesion, survival, and chondrogenic differentiation [25]. Sustained presentation of morphogenic signals such as bone morphogenetic protein-4 (BMP-4) and TGF-β1 promotes extracellular matrix deposition and enhances cartilage tissue formation [26,27]. These combined biochemical and structural properties make heparin-conjugated fibrin hydrogels a promising platform for cell-based cartilage regeneration.

Based on these considerations, we developed an injectable heparin-conjugated fibrin hydrogel (HCFH) designed to serve as a bioactive scaffold for the localized delivery of mesenchymal stromal cells and chondrogenic growth factors [27]. By combining a cell-supportive fibrin matrix with affinity-based growth factor retention mediated by heparin conjugation, this system aims to enhance cartilage regeneration and restore the structural integrity of osteochondral defects.

The aim of the present study was to evaluate the safety, feasibility, and preliminary clinical efficacy of HCFH containing autologous mesenchymal stromal cells and growth factors for the treatment of large osteochondral lesions of the talus.

## 2. Materials and Methods

**Figure 1:**
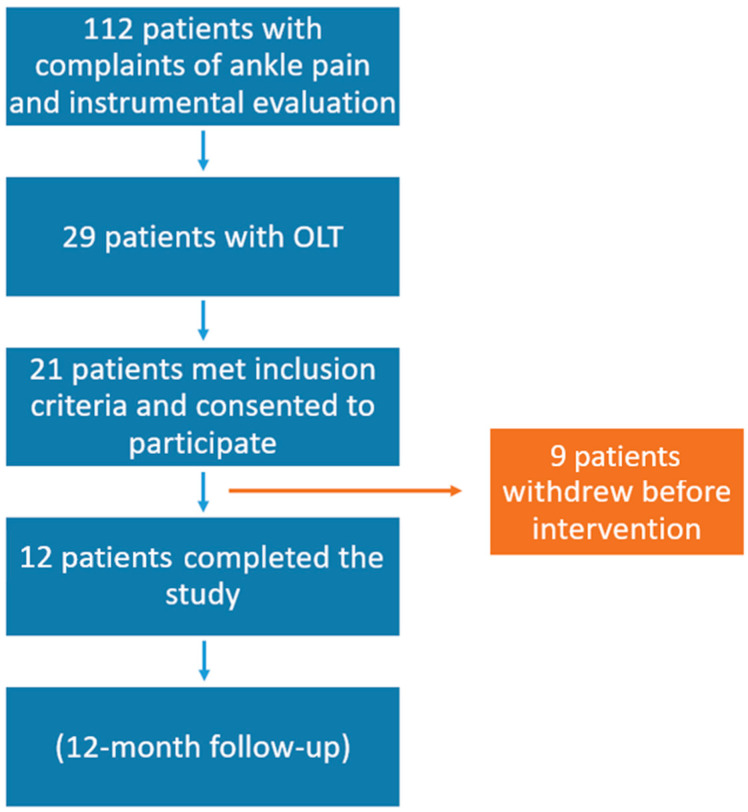
CONSORT flow diagram.

Patients with osteochondral lesions of the talus who satisfied the inclusion criteria were prospectively enrolled in the study. A history of ankle trauma was present in all patients except one. The inclusion criteria comprised an age of up to 65 years inclusive and voluntary informed consent to participate in the study. The exclusion criteria included severe ankle osteoarthritis (Kellgren–Lawrence grade III–IV based on radiographic assessment), synovitis, severe neurocognitive disorders, hemiparesis on the affected side, and the presence of neoplasms with or without metastases, diabetes, systemic inflammatory disease, immunosuppressive conditions, smoking history (Figure 1).

Demographic and baseline characteristics of the study population are presented in Table 1.

The study cohort included predominantly male patients with osteochondral lesions of the talus and a mean age of 36 years. The mean lesion size corresponded to medium-to-large osteochondral defects of the talus.

The study was conducted in compliance with ethical standards in accordance with the Declaration of Helsinki, the Code on Public Health and Healthcare System of the Republic of Kazakhstan dated 7 January 2020, No. 360-VI ZRK, and Order of the Ministry of Health of the Republic of Kazakhstan dated 21 December 2022, No. KR DSM-310/2020 “On the Approval of the Rules for Conducting Biomedical Research and Requirements for Research Centers.” This clinical study was approved by the Local Ethics Committee on Bioethics of the RSE on REM “National Scientific Center of Traumatology and Orthopedics named after Academician N.D. Batpenov” of the Ministry of Health of the Republic of Kazakhstan (protocol No. 4, dated 9 November 2022). The clinical use of autologous mesenchymal stromal cells was performed in accordance with national regulations governing biomedical research. As the cells were autologous and used within the same clinical protocol without genetic modification, no additional regulatory authorization beyond institutional ethical approval was required under current national legislation. The study was registered at ClinicalTrials.gov (NCT06028763). All participants provided written informed consent.

### 2.1. Preoperative Examination

**Figure 2:**
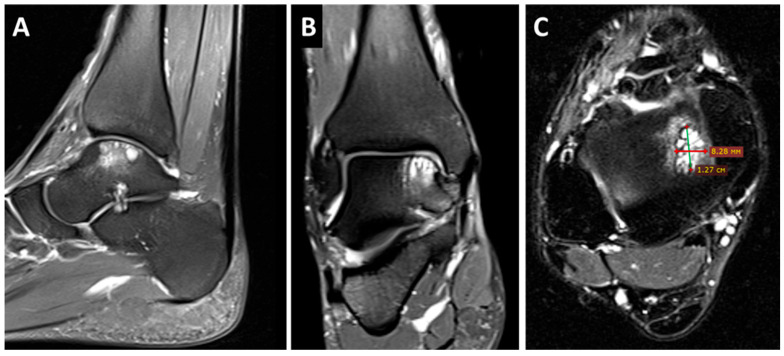
MRI of the osteochondral lesion of the talus (OLT): (**A**)—sagittal view; (**B**)—coronal view; (**C**)—axial view.

Before surgery, all patients underwent magnetic resonance imaging (MRI) to determine the location, shape, and area of the defect, as well as the presence of a free osteochondral fragment (Figure 2).

### 2.2. Adipose Tissue Harvesting for MSCs Isolation

**Figure 3:**
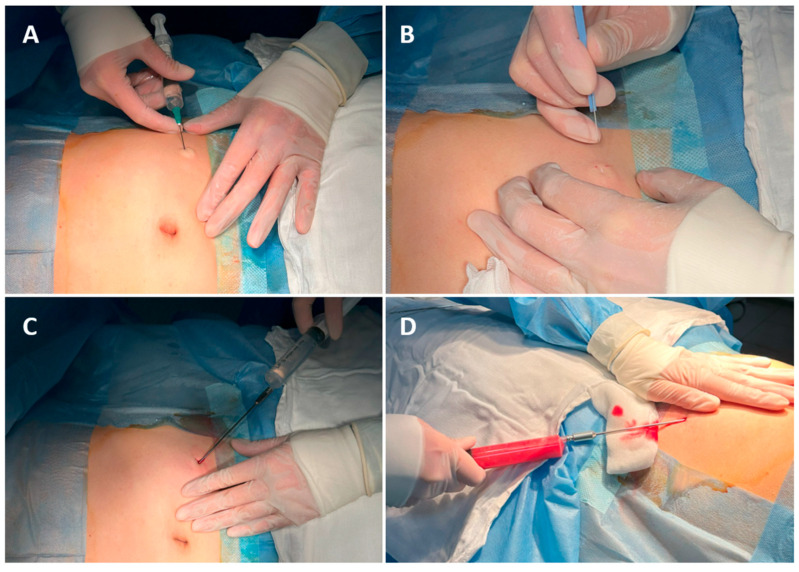
Procedure for harvesting adipose tissue from the anterior abdominal wall: (**A**)—local anesthesia with lidocaine until formation of a “lemon peel” effect; (**B**)—puncture to the subcutaneous fat layer at the donor site; (**C**)—infiltration with Klein’s solution; (**D**)—lipoaspiration.

At the first stage, patients were invited to the operating unit for the collection of autologous adipose tissue required for mesenchymal stromal cell (MSC) isolation. The procedure was performed under local anesthesia. After antiseptic preparation of the anterior abdominal wall, a small skin puncture was made, and Klein’s solution (200–300 mL) was injected into the subcutaneous fat tissue. The composition of the solution was as follows: 400 mL of normal saline, 20 mL of 2% lidocaine solution, 0.4 mL of adrenaline, and 10 mL of sodium bicarbonate (Figure 3).

The solution provided uniform tissue infiltration, facilitating the emulsification of adipose cells. The resulting emulsion was then aspirated using a lipoaspiration cannula and collected into a syringe. The volume solution was selected according to standard tumescent liposuction protocols to ensure uniform tissue infiltration and facilitate adipocyte separation. The ratio between infiltrated solution and aspirated adipose tissue was optimized to maximize stromal vascular fraction yield while maintaining cell viability.

**Figure 4:**
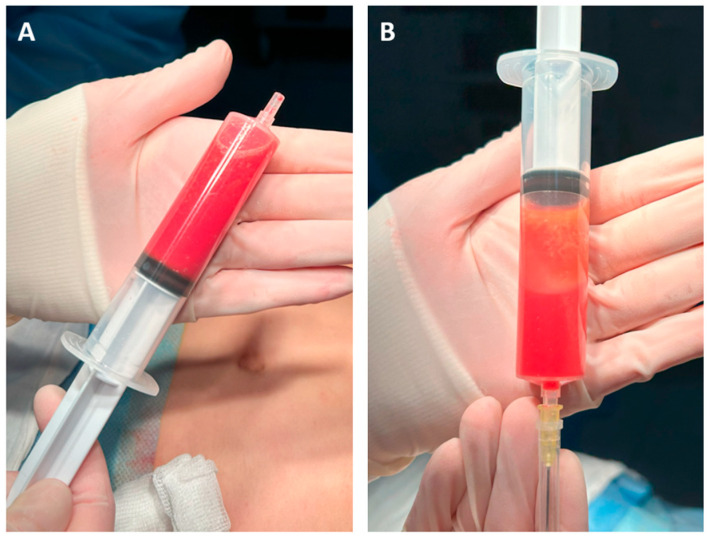
Collected material: (**A**)—lipoaspirate containing admixtures of Klein’s solution and blood; (**B**)—floating adipose tissue during the sedimentation process.

On average, 25–35 mL of lipoaspirate was obtained, which was collected into sterile syringes and transported within the first four hours to the Stem Cell Laboratory of the National Center for Biotechnology LLP (Astana, Kazakhstan) under sterile conditions and maintaining the cold chain (Figure 4).

Under laboratory conditions isolation and cultivation of autologous mesenchymal stromal cells (MSCs) were carried out, as well as the synthesis of HCFH.

### 2.3. Isolation and Cultivation of Human Adipose-Derived MSCs

Lipoaspirate samples were washed with phosphate-buffered saline (PBS) and centrifuged at 400× *g* for 10 min. The stromal vascular fraction was obtained by enzymatic digestion with 0.075% collagenase type I at 37 °C for 45 min and neutralized with α-MEM supplemented with 10% fetal bovine serum (FBS). The stromal vascular fraction yielded approximately (1–5) × 10^5^ nucleated cells per ml of lipoaspirate, with MSCs comprising approximately 1–10% of the total cell population.

Cells were cultured in α-MEM containing 10% FBS and 1% penicillin/streptomycin at 37 °C in a humidified atmosphere with 5% CO_2_. The medium was replaced after 24 h and subsequently every 2–3 days. At 80–90% confluence, cells were detached using TrypLE Express and expanded up to passage 5. Cell number were determined using an automated cell counter (TC20, Bio-Rad, Hercules, CA, USA). All human MSC cultures underwent quality control testing, including sterility assessment for bacterial, yeast and fungal contamination using Cell Culture Contamination Detection Kit (Thermo Fisher Scientific, Burlington, MA, USA), mycoplasma detection by PCR assay kit (Thermo Fisher Scientific), endotoxin testing using the Limulus Amebocyte Lysate (LAL) assay (Charles River Laboratories, Wilmington, MA, USA), and cell viability evaluation by Trypan Blue exclusion. In this study, only MSC cultures that successfully met all predefined release criteria were used for clinical application. Sterility testing confirmed the absence of bacterial, fungal, and yeast contamination, while PCR analysis demonstrated that all cell cultures were negative for mycoplasma. Endotoxin levels were below the established acceptance threshold for clinical use. Cell viability, assessed by the Trypan Blue exclusion assay prior to administration, exceeded 90% in all MSC preparations. These results confirmed compliance of the MSC products with the predefined quality and safety requirements for clinical application.

### 2.4. Flow Cytometric Analysis

The immunophenotypic profile of human adipose-derived MSCs was evaluated by flow cytometer MACSQuant^®^ Analyzer 10 (Miltenyi Biotec, Bergisch Gladbach, Germany) using the Human MSC Analysis Kit (BD Stemflow™, BD Biosciences, San Jose, CA, USA) according to the manufacturer’s instructions. Briefly, 1 × 10^6^ cells per sample were incubated with fluorochrome-conjugated antibodies against positive and negative MSC surface markers, as well as corresponding isotype controls. Following staining, the cells were fixed using Fix/Perm buffer (BD Biosciences). Flow cytometric acquisition was performed on an Attune Flow Cytometer (Thermo Fisher Scientific, Waltham, MA, USA). Dead cells were excluded by 7-AAD staining, and gating was performed based on the characteristic forward- and side-scatter properties of MSCs. Data were analyzed using FlowJo software (BD Biosciences, Ashland, OR, USA).

### 2.5. Colony-Forming Unit-Fibroblast (CFU-F) Assay

The clonogenic potential of isolated cells was assessed using a colony-forming unit-fibroblast (CFU-F) assay. Cells derived from human synovial membrane were seeded into T25 culture flasks at a density of 10 cells/cm^2^ and cultured in complete growth medium for 14 days at 37 °C in a humidified atmosphere containing 5% CO_2_. At the end of the culture period, the cells were washed with phosphate-buffered saline (PBS), fixed, and stained with 0.5% crystal violet solution for 5 min at room temperature. After two washes with PBS, colonies consisting of ≥50 cells were counted under an SZ61 stereomicroscope (Olympus, Hamburg, Germany). Representative images were captured using an SC100 digital camera (Olympus).

### 2.6. Multilineage Differentiation Assays

The multipotent differentiation capacity of the isolated MSCs was evaluated through adipogenic, osteogenic, and chondrogenic induction.

For chondrogenic differentiation, cells were resuspended at a density of 1.25 × 10^6^ cells/mL in chondrogenic induction medium consisting of high-glucose DMEM supplemented with 1% insulin-transferrin-selenium (ITS), 100 μM ascorbate-2-phosphate, 10^−7^ M dexamethasone, and 10 ng/mL recombinant human TGF-β1. Aliquots containing 2.5 × 10^5^ cells were transferred into V-bottom 96-well polypropylene plates (Phoenix, AZ, USA) and centrifuged at 500× *g* to generate cell pellets. Cultures were maintained at 37 °C in a humidified atmosphere containing 5% CO_2_, with medium replacement three times per week. After 21 days, chondrogenic pellets were harvested, fixed in 4% paraformaldehyde (pH 7.2), embedded in paraffin, sectioned, and stained with Toluidine Blue to evaluate cartilaginous matrix formation.

For osteogenic differentiation, cells were cultured for 21 days in osteogenic induction medium containing 10^−7^ M dexamethasone, 10 mM β-glycerophosphate, and 50 μM ascorbate-2-phosphate. Mineralized extracellular matrix deposition was assessed by Alizarin Red S staining.

For adipogenic differentiation, cells were cultured for 21 days in adipogenic induction medium supplemented with 10^−6^ M dexamethasone, 0.5 mM 3-isobutyl-1-methylxanthine (IBMX), and 10 ng/mL insulin. At the end of the induction period, intracellular lipid accumulation was visualized by Oil Red O staining.

### 2.7. Synthesis of Heparin-Conjugated Fibrinogen

The synthesis, physicochemical characterization, growth factor release kinetics, biocompatibility, degradation behavior, and regenerative efficacy of the HCFH has been described previously by our research group [27]. Therefore, only a concise description of hydrogel preparation relevant to the current clinical study is presented herein.

Heparin-conjugated fibrinogen was synthesized according to a previously published method [28]. In total, 100 mg of low-molecular-weight heparin (LMWH; Abcam, Cambridge, UK) was dissolved in 100 mL of 0.05 M 2-(N-morpholino)ethanesulfonic acid (MES) buffer. To activate the carboxyl groups of LMWH, N-hydroxysuccinimide (NHS; 0.04 mM) and N-(3-dimethylaminopropyl)-N′-ethylcarbodiimide hydrochloride (EDC; 0.08 mM) were added, and the reaction mixture was incubated at 4 °C for 12 h. The activated LMWH was then precipitated with excess anhydrous acetone, collected, and lyophilized for 24 h.

Human plasminogen-free fibrinogen (100 mg; Sigma-Aldrich, Burlington, MA, USA) was dissolved in 20 mL phosphate-buffered saline (PBS, pH 7.4) at 4 °C and subsequently reacted with 60 mg of activated lyophilized LMWH for 3 h at 4 °C. The resulting conjugate was precipitated, lyophilized, and dissolved in phosphate-buffered saline (PBS) without Ca^2+^ and Mg^2+^. To remove unconjugated LMWH, the product was dialyzed using dialysis tubing with a molecular weight cut-off of 12,000–14,000 Da at 4 °C for 24 h, followed by lyophilization for 48 h to obtain purified heparin-conjugated fibrinogen. The chemical structure of the lyophilized of heparin-conjugated fibrinogen was characterized using a JNNECA- 500 NMR spectrometer (JEOL, Tokio, Japan) operating at 500 MHz. Spectra were acquired at room temperature in deuterium oxide (D_2_O) as the solvent. The resulting spectra were processed and analyzed using MestReNova software (version 14.2.0; Mestrelab Research, Santiago de Compostela, Spain). Chemical shifts were referenced to the residual HDO signal present in D_2_O and reported in parts per million (ppm). The successful conjugation of heparin to fibrinogen was confirmed by the appearance of characteristic proton resonance signals corresponding to both components in the ^1^H NMR spectra.

For clinical application, the lyophilized heparin-conjugated fibrinogen was sterilized by gamma irradiation at a dose of 15 kGy using an ILU-10 irradiation facility (Park of Nuclear Technologies, Kurchatov, Kazakhstan).

### 2.8. Preparation of HCFH with Human MSCs and Growth Factors

To prepare the HCFH containing human adipose-derived MSCs and growth factors, the fibrinogen and thrombin components were utilized. For preparation of the fibrinogen component, 80 mg of human fibrinogen, 40 mg of heparin-conjugated fibrinogen, 500 ng of recombinant human TGF-β1 (Abcam, UK), and 500 ng of recombinant human BMP-4 (Abcam) aprotinin solution (110 KIU/mL) (Sigma-Aldrich) were dissolved in 1 mL of PBS and incubated at 37 °C for 45 min in a sterile water bath until complete dissolution was achieved. Subsequently, 2 × 10^7^ adipose-derived MSCs were added to the fibrinogen solution with growth factors (1 mL) and gently mixed using a magnetic stirrer for 3 min at 37 °C to ensure uniform cell distribution.

For preparation of the thrombin component, 15 U of human thrombin (Sigma-Aldrich) was reconstituted in 1 mL of 40 μM calcium chloride solution and incubated at 37 °C for 45 min in a sterile water bath. Before preparation of HCFH, aprotinin and thrombin solution were sterilized through the polyethersulfone membrane filter with pore size 0.22 µm (TPP, Trasadingen, Switzerland).

Immediately before implantation, the fibrinogen/cell suspension and thrombin solution were transferred into separate sterile single-use syringes, mounted in a DUPLOJECT dual-syringe applicator, and connected to a DUPLOTIP dual-lumen cannula (20 G × 10 cm; Baxter International Inc., Deerfield, IL, USA). Equal volumes of both components were simultaneously implanted into the cartilage defect, resulting in rapid in situ gelation and formation of the HCFH scaffold containing MSCs and growth factors.

### 2.9. Surgical Procedure

Following preparation of the HCFH, patients underwent the second stage of treatment consisting of ankle arthroscopy, debridement and preparation of the osteochondral defect, followed by implantation of the injectable HCFH. The procedure was performed within approximately one month after adipose tissue harvesting, depending on the time required to obtain the target MSC quantity.

All procedures were carried out under spinal anesthesia using standard arthroscopic equipment. Patients were positioned supine with a pneumatic thigh tourniquet applied to provide a bloodless operative field. Following antiseptic skin preparation and sterile draping, standard anteromedial and anterolateral ankle portals were established.

A systematic arthroscopic inspection of the ankle joint was performed to assess the size and characteristics of the osteochondral lesion and to identify concomitant intra-articular pathology. Loose bodies, when present, were removed arthroscopically, and minor synovial inflammatory changes were treated by debridement. Cases with pronounced synovitis or active inflammatory changes were not considered suitable for implantation.

**Figure 5:**
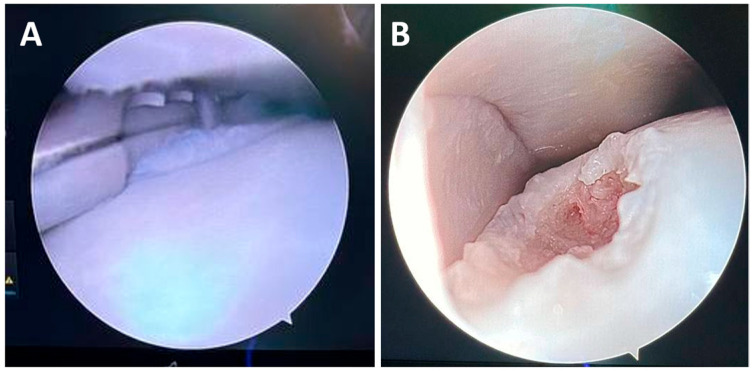
Ankle arthroscopy: (**A**)—identification of the defect area; (**B**)—osteochondral defect after debridement and preparation for HCFH application.

The osteochondral defect was carefully debrided to remove unstable cartilage and the damaged calcified cartilage layer, creating stable vertical margins and a well-defined defect bed suitable for biological reconstruction (Figure 5). Microfracture was subsequently performed using an arthroscopic awl at approximately 3–4 mm intervals and a depth of 2–3 mm to promote communication with the subchondral bone marrow and facilitate the release of marrow-derived progenitor cells.

**Figure 6:**
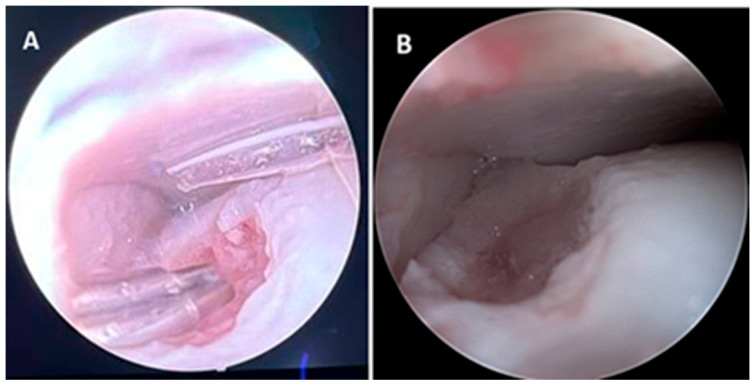
Hydrogel application. (**A**) Introduction of the hydrogel into the defect. (**B**) First hydrogel layer after application.

After completion of defect preparation, the joint cavity was thoroughly dried to ensure optimal HCFH fixation. The fibrinogen/MSC suspension and thrombin component of the HCFH were simultaneously delivered into the defect using a dual-syringe applicator system equipped with a dual-lumen cannula. Mixing of the two components occurred at the time of application, resulting in rapid in situ gelation and formation of a three-dimensional scaffold containing autologous MSCs and growth factors. The defect was completely filled until restoration of the native articular surface contour was achieved (Figure 6).

The implanted hydrogel was allowed to polymerize for approximately 5 min. Final arthroscopic inspection confirmed complete filling of the defect, homogeneous distribution of the implant, and stable fixation within the lesion site. The arthroscopic portals were closed with interrupted sutures, and a sterile dressing was applied.

Postoperatively, patients remained non–weight-bearing on the operated limb for six weeks. Progressive weight-bearing was initiated thereafter according to clinical and radiological findings. All patients participated in a structured rehabilitation program supervised by a rehabilitation physician, focusing on restoration of ankle range of motion, muscle strength, gait recovery, and prevention of postoperative complications.

### 2.10. Assessment of Safety and Presumptive Efficacy

The evaluation of the safety and efficacy of the HCFH was carried out comprehensively, using clinical, instrumental, and laboratory methods, as well as dynamic patient follow-up for 12 months after the intervention.

Safety was evaluated throughout all study stages by clinical inspection of postoperative wounds and laboratory monitoring. Laboratory tests were performed preoperatively, postoperatively, and during follow-up, including complete blood count and serum C-reactive protein (CRP) levels to identify inflammation, infection, or immune reactions to the biomaterial. All adverse events were recorded and graded according to the Common Terminology Criteria for Adverse Events (CTCAE). The severity of complications was classified from Grade I (mild) to Grade V (death). Particular attention was paid to local inflammatory reactions, allergic responses, infection, delayed wound healing, and systemic adverse events.

Efficacy was assessed by analyzing pain intensity and ankle joint function using validated scales: the Visual Analogue Scale (VAS) and the American Orthopedic Foot and Ankle Society (AOFAS) score, recorded preoperatively and at 6 and 12 months after implantation. The Kazakh cross-culturally adapted and validated version of the AOFAS questionnaire was used in this study [29]. AOFAS scores were interpreted as follows: 90–100—excellent; 75–89—good; 50–74—fair; <50—poor outcome.

Morphological evaluation of cartilage repair and subchondral bone was performed using MRI in standard sequences before surgery and at 6 and 12 months postoperatively. MRI images were independently evaluated by two experienced musculoskeletal radiologists blinded to clinical data and follow-up time points. Inter-rater reliability for the modified Magnetic Resonance Observation of Cartilage Repair Tissue (MOCART) score was assessed using Cohen’s kappa coefficient. Cartilage repair quality was assessed using the modified MOCART scoring system [30], interpreted as: 81–100—excellent; 61–80—good; 41–60—fair; ≤40—poor repair. Defect area was calculated as S = π/3 × a × b, where a and b represent maximal defect dimensions in the coronal and sagittal planes.

### 2.11. Statistical Analysis

Data analysis was performed using SPSS software, version 26.0 (IBM Corp., Armonk, NY, USA). Quantitative variables were tested for normality using the Shapiro–Wilk test. Variables with a normal distribution were described as mean (M) ± standard deviation (SD) and 95% confidence interval (95% CI). For non-normally distributed data, results were presented as median (Me) and interquartile range (Q1–Q3). Qualitative variables were expressed as absolute frequencies and percentages. For comparative analysis, the paired Student’s t-test was applied to normally distributed variables, whereas the Wilcoxon signed-rank test was used for non-normally distributed paired data.

## 3. Results

### 3.1. Characterization of Human Adipose-Derived MSCs

**Figure 7:**
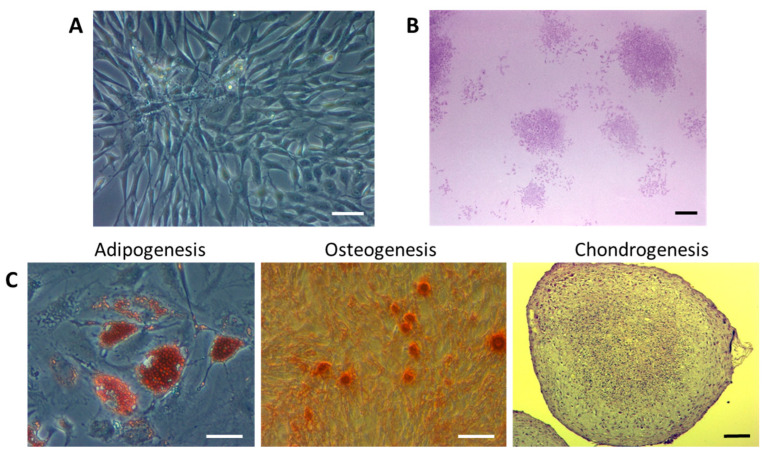
Characterization of human adipose-derived MSCs: (**A**)—Phase-contrast image showing the morphology of adipose-derived MSCs in culture (scale bar: 20 µm); (**B**)—Representative image of colony-forming unit-fibroblast (CFU-F) colonies obtained after 14 days from passage 2 MSC cultures (scale bar: 1 mm); (**C**)—Multilineage differentiation potential of MSCs. Adipogenic differentiation was confirmed by Oil Red O staining, osteogenic differentiation by Alizarin Red staining, and chondrogenic differentiation by Toluidine Blue staining (scale bars: 50 µm for adipogenic and osteogenic staining; 100 µm for chondrogenic staining).

Human adipose-derived MSCs exhibited a typical fibroblast-like spindle-shaped morphology and formed a homogeneous adherent cell population during in vitro expansion (Figure 7A). The cells demonstrated robust proliferative activity and clonogenic potential, as confirmed by the colony-forming unit-fibroblast (CFU-F) assay. Numerous well-defined colonies were observed after 14 days of culture, indicating the presence of progenitor cells with self-renewal capacity (Figure 7B).

The multipotent differentiation potential of adipose-derived MSCs was confirmed by successful adipogenic, osteogenic, and chondrogenic differentiation. Adipogenic induction resulted in the accumulation of intracellular lipid droplets visualized by Oil Red O staining. Osteogenic differentiation was evidenced by the formation of mineralized extracellular matrix deposits positively stained with Alizarin Red S. Chondrogenic differentiation produced compact cell pellets containing cartilage-like extracellular matrix, as demonstrated by positive Toluidine Blue staining (Figure 7C). These findings confirmed the multilineage differentiation capacity characteristic of mesenchymal stromal cells.

**Figure 8:**
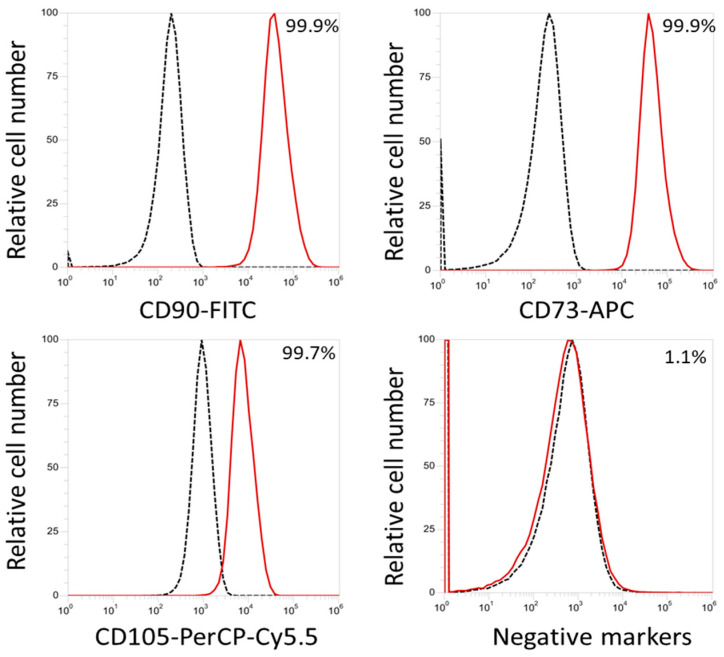
Phenotypic characterization of human adipose-derived MSCs by flow cytometry. Flow cytometric immunophenotyping of human adipose-derived MSCs. Representative histograms demonstrate the expression of positive (CD90, CD73, CD105) and negative MSC surface markers (CD34, CD45, CD11b, CD19, HLA-DR) (red) relative to corresponding isotype controls (black). Dead cells were excluded by 7-AAD staining, and analysis was performed on the viable cell population gated according to the characteristic light-scattering properties of MSCs.

Flow cytometric analysis further confirmed the mesenchymal phenotype of the cultured cells. adipose-derived MSCs showed high expression of the positive MSC surface markers CD90 (99.9%), CD73 (99.9%), and CD105 (99.7%). In contrast, expression of hematopoietic and immune-associated markers (CD34, CD45, CD11b, CD19, and HLA-DR) was minimal, with a combined positivity of only 1.1% (Figure 8). The immunophenotypic profile was consistent with the minimal criteria established by the International Society for Cell & Gene Therapy for the identification of MSCs.

Collectively, morphological assessment, clonogenicity testing, trilineage differentiation assays, and flow cytometric immunophenotyping confirmed the successful isolation and expansion of a highly purified population of human adipose-derived MSCs suitable for incorporation into the HCFH and subsequent clinical application.

### 3.2. Clinical Outcomes

In total, 12 patients with diagnosed osteochondral defects of the talus were included in the study.

Inflammatory markers. Postoperative wound healing was evaluated by the presence of pain, erythema, necrosis, or purulent discharge. Primary healing was defined as intact suture lines without inflammation or necrosis at discharge, whereas secondary healing indicated marginal necrosis or erythema. All patients demonstrated primary wound healing without infectious or allergic complications. According to CTCAE v5.0 criteria, no Grade III–V adverse events were observed during the 12-month follow-up. All recorded postoperative reactions (including transient local edema and laboratory inflammatory marker elevation) were classified as Grade I and resolved spontaneously without specific treatment. Laboratory analysis revealed a transient postoperative increase in inflammatory markers, which decreased substantially by day 14 and were not associated with clinical signs of infection (Table 2).

**Figure 9:**
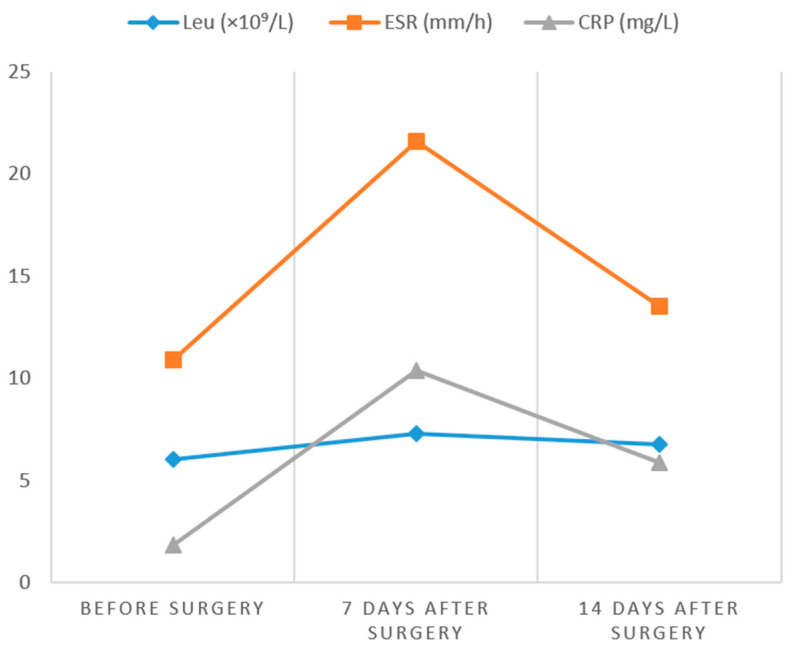
Dynamics of laboratory inflammatory markers before and after surgery.

Laboratory analysis demonstrated a transient postoperative increase in inflammatory markers during the early postoperative period, followed by substantial normalization by day 14 (Table 2). The temporal dynamics of laboratory inflammatory markers are illustrated in Figure 9. The observed elevations in leukocyte count, ESR, and CRP were consistent with the expected physiological response to surgery and were not associated with clinical signs of infection or systemic complications. All laboratory parameters showed a clear downward trend during follow-up, supporting the safety of the procedure and the absence of pathological inflammation.

The postoperative inflammatory response was transient and consistent with the expected physiological reaction to surgery, without evidence of infection or systemic complications. Short-term increases in CRP, ESR, and leukocyte counts normalized by day 14, confirming the absence of pathological inflammation [31,32].

Functional outcome analysis demonstrated a significant and sustained improvement in both pain intensity and ankle joint function during the 12-month follow-up. According to the Visual Analogue Scale (VAS), median pain scores decreased from 6.0 [5.52; 6.48] preoperatively to 3.0 [2.67; 3.23] at 6 months (*p* < 0.001) and further to 2.0 [1.41; 2.09] at 12 months (*p* < 0.001). Correspondingly, the American Orthopedic Foot and Ankle Society (AOFAS) score improved from 70.0 [64.16; 71.44] before surgery to 83 [77.58; 83.52] at 6 months (*p* < 0.001) and 90.6 ± 4.57 at 12 months (*p* < 0.001).

These findings indicate a clinically and statistically significant improvement in functional outcomes, characterized by progressive pain reduction and restoration of ankle joint mobility and function within one year following HCFH application (Table 3).

#### MRI Assessment (MOCART Score)

**Figure 10:**
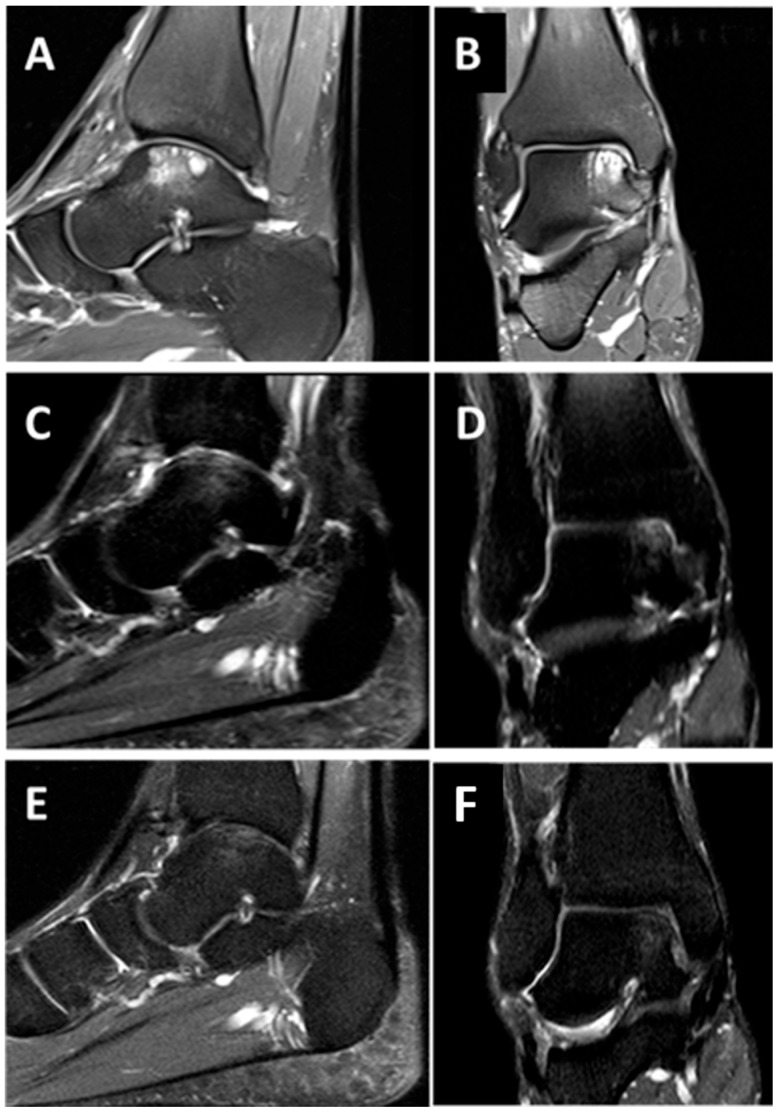
Ankle MRI in a patient with an osteochondral lesion of the talus (OLT): preoperative: (**A**) sagittal view; (**B**) coronal view; 6 months postoperatively: (**C**) sagittal view; (**D**) coronal view; 12 months postoperatively: (**E**) sagittal view; (**F**) coronal view.

MRI evaluation using the modified MOCART scoring system demonstrated progressive structural maturation of the repair tissue between 6 and 12 months (Figure 10). Interobserver agreement between the evaluators was substantial to almost perfect for most MRI parameters, with Cohen’s κ values ranging from 0.68 (95% CI 0.27–1.00) to 0.84 (95% CI 0.54–1.00). Perfect agreement (100%) was observed for surface assessment, bony defect/overgrowth, and cysts; therefore, Cohen’s kappa could not be calculated for these variables due to the absence of variability in ratings (Table 4).

Perfect agreement between evaluators; Cohen’s κ was not calculated due to lack of variability.

At 6 months postoperatively, the mean MOCART score in the study group was 34.16 ± 17.1, reflecting the presence of immature reparative tissue with partial filling of the defect and limited integration with the surrounding cartilage. By 12 months, the median MOCART score had significantly increased to 75 ± 5.43 (*p* < 0.001), indicating near-complete defect filling, improved surface congruity, and integration with adjacent cartilage (Table 5).

The signal intensity of the repair tissue approached that of the surrounding native cartilage in most cases, although subtle differences persisted in some patients. These findings were consistent with cartilage-like tissue formation rather than definitive confirmation of hyaline cartilage regeneration.

The observed improvements were statistically significant and were accompanied by MRI findings consistent with progressive cartilage-like tissue formation over the 12-month follow-up period. However, the study did not include second-look arthroscopy, and therefore direct comparison with other treatment modalities or histological confirmation of tissue quality was not performed.

## 4. Discussion

In this prospective single-group study, arthroscopically assisted implantation of injectable HCFH for osteochondral lesions of the talus demonstrated significant clinical and morphological improvement at 12 months. Pain intensity decreased from 6.0 to 2.0 (VAS), while ankle function improved from 70.0 to 90.6 (AOFAS, *p* < 0.001). MRI assessment showed a rise in MOCART score from 34.16 ± 17.1 at 6 months to 75 ± 5.43 at 12 months (*p* < 0.001), indicating complete defect filling and satisfactory tissue integration. Transient postoperative elevation of inflammatory markers normalized by day 14, confirming a physiological response without infection. All wounds healed by primary intention, supporting the hydrogel’s safety and biocompatibility.

These findings are consistent with previously reported outcomes of surgical treatment for osteochondral lesions of the talus. In studies evaluating bone marrow stimulation (BMS), mean AOFAS scores increased from 62.4 ± 7.9 preoperatively to 83.9 ± 9.2 at a mean follow-up of 54.1 months [33], whereas cartilage restoration procedures such as ACI/MACI, AMIC, and OATS typically demonstrate postoperative AOFAS values ranging from 84 to 94 points [13,34,35,36,37,38]. Similarly, pain reduction reported in the literature shows decreases in Visual Analog Scale scores from approximately 6–8 preoperatively to 0–2 postoperatively, which is comparable to the improvement observed in the present cohort [13,34,35,36,37,39,40,41].

However, MRI-based structural assessment often demonstrates only moderate cartilage repair despite favorable clinical outcomes. Meta-analyses report mean MOCART score values of approximately 63.9 ± 15.5 after bone marrow stimulation [42], while cartilage restoration procedures such as ACI/MACI and AMIC typically yield scores ranging from 50 to 65 points [35,36,37]. In contrast, the MOCART score observed in the present study at one year was higher than that reported in most previous studies, suggesting favorable early morphological repair. This finding may be attributed to the biological properties of the HCFH, which provides a stable scaffold facilitating cell migration and extracellular matrix deposition, thereby potentially enhancing early cartilage regeneration. Heparin conjugation may additionally contribute to improved hydrogel stability and scaffold integrity [43]. Covalent incorporation of heparin into the fibrin network can enhance intermolecular interactions and facilitate prolonged retention of growth factors involved in extracellular matrix synthesis and tissue remodeling [44]. Although fibrin hydrogels are generally characterized by limited mechanical strength in load-bearing environments, the contained nature of the osteochondral defect together with postoperative non–weight-bearing rehabilitation likely supported early scaffold stabilization and tissue maturation [45,46]. Nevertheless, further biomechanical studies are needed to evaluate the long-term structural durability of the regenerated tissue.

The observed improvement in MOCART scores is supported by preclinical evidence demonstrating that HCFH provides structural support, sustained morphogen release, and an optimal regenerative microenvironment. In experimental rabbit models, co-delivery of synovium-derived mesenchymal stromal cells together with TGF-β1 and BMP-4 within an HCFH resulted in superior restoration of hyaline-like cartilage and subchondral bone compared with treatments using cells or growth factors alone [27]. Clinical evidence also supports the regenerative potential of this approach. In a study including 38 patients with focal cartilage defects of the knee, treatment using a similar heparin-conjugated fibrinogen -based system demonstrated favorable two-year outcomes, including pain reduction, functional improvement, and partial cartilage restoration in the majority of cases [47]. Nevertheless, as the present findings reflect only short-term follow-up, further long-term randomized controlled trials are required to determine the durability and long-term structural stability of the regenerated tissue.

Treatment selection for OLT depends on lesion size, depth, and morphology. BMS techniques—including drilling, microfracture, and abrasion arthroplasty—are designed to penetrate the subchondral plate and stimulate the release of progenitor cells and growth factors that promote cartilage repair [33,48,49]. These procedures are generally recommended for contained defects ≤10–15 mm or ≤150 mm^2^ and have demonstrated favorable short- and mid-term clinical outcomes. However, the repair tissue formed after BMS is predominantly fibrocartilage, which possesses inferior biomechanical properties compared with native hyaline cartilage and may deteriorate over time [50,51,52]. For larger or recurrent lesions, osteochondral autograft transplantation (OATS) represents an alternative treatment strategy and has been associated with durable clinical outcomes. Nevertheless, this technique is limited by potential donor-site morbidity, restricted graft availability, and the frequent need for malleolar osteotomy to access the talar dome [9,53,54].

Cell-based therapies like autologous chondrocyte implantation (ACI) and matrix-induced ACI (MACI) achieve hyaline-like repair in 80–90% of medium-sized defects [55,56,57,58,59,60,61]. However, these approaches are associated with high treatment costs, a two-stage surgical procedure, and the need for specialized laboratory processing. Although the proposed MSC-based strategy also requires laboratory cell processing, it may offer several practical advantages, including minimally invasive adipose tissue harvesting under local anesthesia without the need for additional hospitalization, as well as avoidance of a separate cartilage harvesting procedure typically required for ACI/MACI.

Consequently, regenerative approaches employing mesenchymal stromal cells (MSCs) and biomaterial scaffolds are gaining attention [14,15,16,42]. Among MSC sources, adipose tissue is optimal due to accessibility and high cell yield (up to 10% of nucleated cells) [62]. Hydrogels, particularly fibrin- and collagen-based, offer a three-dimensional microenvironment that preserves cell viability and supports chondrogenic differentiation [63,64]. Hydrogels are widely used in cartilage tissue engineering because of their high water content, porous structure, and ability to mimic the native extracellular matrix microenvironment. These properties support cell adhesion, migration, proliferation, and chondrogenic differentiation while also enabling localized and sustained delivery of bioactive molecules. Injectable hydrogel systems additionally allow minimally invasive implantation and adaptation to irregularly shaped cartilage defects. Among available biomaterials, fibrin-based hydrogels have been widely investigated because of their favorable biological properties and ability to support cartilage regeneration [65,66]. Heparin-conjugated fibrin hydrogels may further enhance this regenerative potential by binding and gradually releasing growth factors, including TGF-β and BMPs, thereby promoting cartilage repair and extracellular matrix formation [27]. The single-stage HCFH technique thus offers a practical bridge between traditional BMS and complex cell-based reconstructions. It combines biological stimulation with minimally invasive arthroscopy, potentially providing a cost-effective, accessible treatment for small-to-large OLTs.

This study has several limitations that should be acknowledged. First, its single-arm design without a control or comparator group limits the ability to attribute clinical improvements solely to the HCFH intervention and precludes direct comparison with established techniques such as BMS or AMIC. Second, the relatively small sample size reduces statistical power, may limit the generalizability of the findings, and precluded meaningful subgroup analyses (e.g., according to lesion size, age, or sex). Therefore, potential differences in treatment efficacy among specific patient subgroups could not be explored. Third, the follow-up duration of 12 months restricts conclusions regarding the long-term durability of the reparative tissue and the risk of deterioration or reintervention over time. Additionally, the study did not include histological assessment of the repair tissue; therefore, the structural and biochemical quality of the regenerate could not be verified beyond MRI-based evaluation. Furthermore, MSC expansion was performed using fetal bovine serum (FBS)-supplemented culture medium. Although no treatment-related allergic, inflammatory, or immunological adverse reactions were observed, the use of animal-derived culture supplements may limit the translational and regulatory applicability of the proposed therapeutic approach. Future studies should incorporate xeno-free or human platelet lysate-based culture systems to enhance clinical translation and regulatory compliance. Although a standardized rehabilitation protocol was used, adherence to postoperative restrictions could not be fully controlled, potentially affecting the quality of cartilage repair. Future multicenter randomized controlled trials with larger cohorts, long-term follow-up, and advanced imaging and histological analyses are warranted to validate these preliminary findings and determine the comparative effectiveness of HCFH relative to established cartilage repair techniques. An additional limitation concerns patient attrition. Of the initially consented cohort, eight patients did not complete the 12-month follow-up, primarily due to loss to follow-up, relocation, or personal reasons unrelated to adverse events. No withdrawals were associated with treatment-related complications. Nevertheless, this attrition rate may introduce selection bias, potentially overestimating treatment effects. Furthermore, all procedures were performed by an experienced orthopedic surgeon trained in arthroscopic cartilage repair techniques. While this ensured technical consistency, it may limit the generalizability of outcomes to centers with varying levels of surgical expertise, representing a potential source of performance bias.

Given the 12-month follow-up inherent to this study, conclusions regarding long-term durability cannot yet be drawn. Extended follow-up at 60 months is currently planned to evaluate structural stability of the repair tissue, maintenance of functional improvement, and potential late complications or reinterventions.

In summary, within the limitations of this single-arm study, arthroscopic implantation of HCFH demonstrated an acceptable safety profile and favorable preliminary clinical and radiological outcomes at 12 months. While these findings suggest regenerative potential, larger controlled trials with long-term follow-up are required to confirm comparative efficacy and durability.

## 5. Conclusions

Within the limitations of this single-arm study, arthroscopic application of injectable heparin-conjugated fibrin hydrogel (HCFH) demonstrated an acceptable safety profile and favorable preliminary clinical and radiological outcomes at 12 months. Significant improvements in pain and ankle function were observed, accompanied by MRI findings consistent with cartilage-like tissue formation. These findings suggest preliminary clinical potential of this single-stage regenerative approach for patients with osteochondral lesions of the talus across a range of defect sizes. However, larger controlled studies with extended follow-up are required to confirm comparative efficacy, long-term durability, and broader applicability.

## Figures and Tables

**Table 1:** Demographic and baseline characteristics of patients.

Parameter	Study Group Mean ± SD/% (*n*)
Age (years)	36.08 ± 10.33
Sex	
Male	67% (8)
Female	33% (4)
Weight (kg)	79.58 ± 21.24
Height (cm)	170.25 ± 7.87
BMI (kg/m^2^)	27.17 ± 4.44
Affected side	
Right	58% (7)
Left	42% (5)
Lesion size (mm^2^)	80.4 ± 50.42

**Table 2:** Laboratory parameters of the patients at different stages of observation.

	Leu (×10^9^/L)	ESR (mm/h)	CRP (mg/L)
Before surgery	6.04 ± 1.03	9.0 [5.84; 15.96]	1.55 [1; 3.01]
*p*-value (before/7 days)	0.003	<0.001	0.003
After 7 days	7.31 ± 1.43	20.5 [16.6; 26.6]	10.25 [6.47; 14.34]
*p*-value (before/14 days)	0.19	0.03	0.004
After 14 days	6.21 [5.96; 7.55]	10.5 [9.65; 17.35]	5.05 [3.89; 7.91]

Leu—leukocytes; ESR—erythrocyte sedimentation rate; CRP—C-reactive protein. Data are presented as mean ± standard deviation (SD) and median [Q1; Q3], where Q1 and Q3 denote the first and third quartiles.

**Table 3:** Dynamics of efficacy results according to VAS and AOFAS scales.

	VAS	AOFAS
Before surgery	6.0 [5.52; 6.48]	70 [64.16; 71.44]
*p*-value (before/after 6 months)	<0.001	<0001
After 6 months	3.0 [2.67; 3.23]	83 [77.58; 83.52]
*p*-value (before/after 12 months)	<0.001	<0.001
After 12 months	2.0 [1.41; 2.09]	90.6 ± 4.57

VAS—visual analogue scale (pain); AOFAS—American Orthopedic Foot and Ankle Society score. Data are presented as mean ± standard deviation (SD) and median [Q1; Q3], where Q1 and Q3 denote the first and third quartiles.

**Table 4:** Interobserver reliability of MRI evaluation parameters assessed using Cohen’s kappa coefficient.

№	Variables MOCART 2.0 Ankle Score	Cohen’s κ	95% CI
1	Volume fill	0.72	0.38–1.00
2	Integration	0.84	0.54–1.00
3	Surface	100% agreement *	
4	Signal intensity	0.68	0.27–1.00
5	Bony defect/overgrowth	100% agreement *	
6	Edema-like marrow signal	0.82	0.49–1.00
7	Cysts	100% agreement *	

* Complete agreement between observers was observed; Cohen’s κ coefficient was not calculated due to the absence of variability in the ratings. MOCART—magnetic resonance observation of cartilage repair tissue.

**Table 5:** Dynamics of cartilage repair according to MOCART score.

	MOCART Score
After 6 months	34.16 ± 17.1
After 12 months	75 ± 5.43
Intragroup analysis	*p* < 0.001

MOCART—magnetic resonance observation of cartilage repair tissue.

## Data Availability

The data of this study is available from the corresponding author, Meruyert Makhmetova, upon reasonable request.

## Abbreviations

The following abbreviations are used in this manuscript:

OA	Osteoarthritis
PRISMA	Preferred Reporting Items For Systematic Reviews And Meta-Analyses
VAS	Visual Analogue Scale
AOFAS	American Orthopaedic Foot & Ankle Society Scale
RCT	Randomized Control Trial
HA	Hyaluronic Acid
NSAID	Nonsteroidal Anti-Inflammatory Drugs
MRI	Magnetic Resonance Imaging
HCFH	Heparin-Conjugated Fibrin Hydrogel
